# Ethylene-Mediated Drought Tolerance in the Critically Endangered *Artocarpus nanchuanensis*: Insights from Physiological and Transcriptomic Analyses

**DOI:** 10.3390/plants14172636

**Published:** 2025-08-24

**Authors:** Zhe Zhang, Yunli Chen, Fang Yang, Kunjian Yang, Wenqiao Li, Xiao Zhang, Wanhong Liu, Hongping Deng

**Affiliations:** 1 Key Laboratory of Eco-Environment in the Three Gorges Reservoir Region, Ministry of Education, School of Life Sciences, Southwest University, Chongqing 400715, China; swfu08zz@163.com (Z.Z.); cylmail0524@163.com (Y.C.); liwenqiao126@163.com (W.L.); zh_xiao19@163.com (X.Z.); 2 School of Chemistry and Chemical Engineering, Chongqing University of Science and Technology, Chongqing 401331, China; fyang@cqust.edu.cn (F.Y.); kjyang@cqust.edu.cn (K.Y.); whliu@cqust.edu.cn (W.L.)

**Keywords:** critically endangered species, *Artocarpus nanchuanensis*, drought stress, ethephon, physiological and molecular responses

## Abstract

Drought stress limits seedling growth, hindering morphological development and population establishment. *Artocarpus nanchuanensis*, a critically endangered species endemic to the karst regions of southwest China, exhibits poor population structure and limited natural regeneration in the wild, with water deficit during the seedling stage identified as a major factor contributing to its endangered status. Elucidating the physiological and molecular mechanisms underlying drought tolerance in *A. nanchuanensis* seedlings is essential for improving their drought adaptability and facilitating population recovery. In this study, 72 two-year-old seedlings were divided into two groups: drought (PEG) and ethephon (PEG + Ethephon), and subjected to drought-rehydration experiments. The results showed that exogenous application of 100 mg·L^−1^ ethephon significantly improved stomatal conductance and photosynthetic pigment content in *A. nanchuanensis* seedlings. Under drought stress, the PEG + Ethephon group exhibited rapid stomatal closure, maintaining water balance and higher photosynthetic pigment levels. After rehydration, the PEG + Ethephon group significantly outperformed the PEG group in terms of photosynthetic rate. Ethephon treatment reduced H_2_O_2_ and MDA levels, enhanced antioxidant enzyme activity (SOD, CAT, POD, GR), and increased osmotic regulator activity (soluble sugars, soluble proteins, and proline), improving ROS-scavenging capacity and reducing oxidative damage. Ethephon application significantly enhanced ethylene accumulation in seedlings, while drought stress stimulated the concentrations of key ethylene biosynthetic enzymes (SAMS, ACS, and ACO), thereby further contributing to improved drought resistance. Transcriptomic data revealed that drought stress significantly upregulated key ethylene biosynthesis genes, with expression levels increasing with stress duration and rapidly decreasing after rehydration. WGCNA analysis identified eight key drought-resistance genes, providing valuable targets for future research. This study provides the first mechanistic insight into the physiological and molecular responses of *A. nanchuanensis* seedlings to drought and rehydration, underscoring the central role of endogenous ethylene in drought tolerance. Ethephon treatment effectively enhanced ethylene accumulation and biosynthetic enzyme activity, thereby improving drought adaptability. These findings lay a theoretical foundation for subsequent molecular functional studies and the conservation biology of this endangered species.

## 1. Introduction

Drought is recognized as one of the most severe abiotic stresses currently affecting plant growth and survival, and its global prevalence is expected to intensify under ongoing climate change [1,2]. The availability of water is fundamental to all stages of the plant life cycle, including seed germination, vegetative and reproductive growth, and dormancy maintenance. China is among the countries most frequently impacted by drought, where the increasing occurrence of extreme weather events has further exacerbated the spatial and temporal variability of precipitation [3,4]. Against the backdrop of increasing global drought events, elucidating the physiological and molecular mechanisms underlying drought responses in endangered plant species across different developmental stages is crucial for understanding their adaptive strategies and evolutionary potential.

Ethylene biosynthesis in plants originates from methionine, proceeding through the intermediates S-adenosylmethionine (SAM) and 1-aminocyclopropane-1-carboxylic acid (ACC), and is primarily catalyzed by ACC synthase (ACS) and ACC oxidase (ACO). Ethylene signaling plays a central role in mediating plant perception and response to drought stress. This pathway, which has been extensively studied in the model dicot *Arabidopsis thaliana*, is initiated by membrane-bound ethylene receptors such as ETR1, ETR2, ERS1, ERS2, and EIN4 and transmits signals through a cascade of downstream components to regulate the expression of drought-responsive genes [5,6]. Under drought conditions, the expression of ACS and ACO is commonly upregulated, leading to increased ethylene production and enhanced drought responsiveness. Experimental evidence suggests that key genes in the ethylene biosynthesis pathway are significantly induced under drought stress, contributing to ethylene accumulation that facilitates stomatal closure and root growth, thereby improving plant drought tolerance [7].

Ethephon (2-chloroethylphosphonic acid) is a widely used plant growth regulator that functions as a slow-releasing ethylene donor [8]. Owing to its high permeability, stable ethylene release, and ease of absorption, it has been extensively applied in studies of plant stress physiology and fruit ripening. Recent evidence shows that exogenous ethephon markedly enhances plant drought tolerance by modulating physiological and biochemical processes related to drought adaptation [9]. Ethephon application can increase the activity and expression of ethylene biosynthetic enzymes, thereby elevating ethylene levels. While drought stress alone induces ethylene biosynthesis, exogenous ethylene further regulates this process [10]. In maize, ethephon upregulates ACS and ACO expression under drought, whereas in tomato, although ACS and ACO transcript levels show limited change, other ethylene-biosynthesis-related genes are strongly induced [9,11]. In woody plants, Populus overexpressing *PtoACO7* exhibit higher leaf water potential and lower MDA content under drought, providing clear evidence that the “ACO–ROS” module enhances drought tolerance [12]. Collectively, these findings indicate that ethephon regulates ethylene biosynthesis at the transcriptional level and effectively improves plant drought resistance.

*Artocarpus nanchuanensis*, a narrowly distributed and highly endangered tree species of the subgenus *Pseudojaca* (family Moraceae), is endemic to the karst regions of Chongqing, China. Fewer than 50 mature individuals remain in the wild [13], leading to its designation as a National Class II Protected Plant (List of National Key Protected Wild Plants, 2021) and its classification as “Critically Endangered (CR)” in the China Biodiversity Red List—Higher Plants (2020). As the northernmost naturally distributed species of the predominantly tropical genus *Artocarpus* [14], it holds significant scientific value for evolutionary and ecological studies. Although *A. nanchuanensis* produces seeds abundantly and seedlings are often found near parent trees, the absence of juveniles indicates a potential bottleneck in natural regeneration. Soil moisture is a key factor influencing seed germination and seedling establishment [5]. In recent years, the Chongqing region has experienced increasingly frequent droughts and heatwaves due to climate change, posing serious challenges to seedling survival—particularly in karst areas with shallow soils and uneven water availability [15]. These observations suggest that drought stress may be a major abiotic constraint limiting seedling recruitment and population recovery. Given the species’ high extinction risk, urgent conservation-focused research is essential to enhance seedling survival, promote regeneration, and ensure long-term species persistence.

In this study, drought and rehydration experiments were conducted on *A. nanchuanensis* seedlings to comprehensively assess drought-related physiological parameters and perform transcriptomic analysis, revealing the drought response mechanisms of *A. nanchuanensis*. The results confirmed that endogenous ethylene plays a critical role in regulating drought tolerance in *A. nanchuanensis* seedlings, and exogenous application of 100 mg·L^−1^ ethephon significantly enhanced their drought resistance. This conclusion provides new insights into the drought tolerance strategies of critically endangered woody plants and lays a solid foundation for understanding their adaptive mechanisms, enhancing stress resistance, and promoting future conservation and utilization.

## 2. Materials and Methods

### 2.1. Plant Materials and Experimental Conditions

Prior to drought treatment, all *A. nanchuanensis* seedlings were grown in a controlled environment growth chamber (PG034 Reach, St. Louis, MO, USA) for three months, with conditions set at a temperature of 20–25 °C, a 16 h light/8 h dark photoperiod, and a light intensity of 15,000 Lux. After acclimatization, 72 healthy seedlings with uniform growth and no signs of disease or pest infestation were selected. All seedlings were thoroughly watered, and excess moisture was allowed to drain from the soil. To ensure the consistency and stability of drought conditions, this study used PEG 6000 to simulate drought stress. On the following day, one group of seedlings (36 individuals, PEG + Ethephon group) was foliar-sprayed with ethephon at a concentration of 100 mg·L^−1^ (purchased from China Sichuan Runer Technology Co., Ltd., Chengdu, China, product concentration 480 g·L^−1^) [16]. Spraying was performed until the leaf surface was fully covered with droplets without dripping. The control group was sprayed with distilled water using the same method. Spraying was conducted at 11:00 am for three consecutive days [17].

Subsequently, drought stress was simulated by irrigating the plants with a 10% (*w*/*v*) PEG 6000 solution (36 individuals, PEG group), and the day following PEG application was designated as day 0 of drought stress [18]. For the rewatering experiment, plants subjected to drought for four days were irrigated continuously with pure water for 1 min to thoroughly flush out residual PEG solution from the soil.

During drought treatment, the morphology of the *A. nanchuanensis* seedlings was observed. Leaf samples were collected from the second fully expanded leaf from the apex of each seedling at four time points: day 0, day 2, and day 4 of drought stress and day 2 after rewatering. Samples were immediately frozen in liquid nitrogen and stored at −80 °C until analysis. Each treatment at each time point included three biological replicates, with each replicate consisting of three individual seedlings, resulting in a total of nine seedlings per time point.

### 2.2. Scanning Electron Microscopy of Leaf Stomata

Fresh leaf tissues (≤3 mm^2^) were excised with a sharp blade to minimize mechanical damage, immediately fixed in electron microscopy fixative at room temperature for 2 h, and then stored at 4 °C. Samples were washed three times with 0.1 M phosphate buffer (pH 7.4), post-fixed in 1% OsO_4_ for 1–2 h, and washed again with buffer [19]. After fixation, tissues were dehydrated through an ethanol gradient (30–100%, 15 min each) and treated with isoamyl acetate for 15 min. Dehydrated samples were dried using a critical point dryer (Quorum K850, Quorum Technologies, Laughton, UK), mounted on stubs with carbon tape, sputter-coated with gold (HITACHI, MC1000, Hitachi High-Technologies Corporation, Tokyo, Japan), and imaged using a scanning electron microscope (HITACHI, SU8200, Hitachi High-Technologies Corporation, Tokyo, Japan).

### 2.3. Determination of Leaf Relative Water Content (RWC)

Leaf RWC was measured using the gravimetric method. Fresh leaf samples (1–1.5 g) were collected and immediately weighed to record the fresh weight (FW). The samples were then placed in distilled water in the dark at room temperature for 4–6 h to reach full turgidity. After the surface moisture was blotted with filter paper, the turgid weight (TW) was recorded. Finally, the samples were dried in an oven (Memmert, UFP800, Memmert GmbH + Co. KG, Schwabach, Germany) at 75 °C for 24 h to determine the dry weight (DW).

### 2.4. Determination of Antioxidant Enzyme Activities

About 0.1 g of leaf tissue was ground in 10 mL of 0.05 M phosphate buffer (pH 7.8) on ice. The homogenate was then centrifuged at 12,000 rpm for 20 min at 4 °C (Eppendorf 5810 R, Eppendorf, Germany), and the supernatant was carefully transferred to a fresh centrifuge tube for enzyme activity analysis.

The activities of superoxide dismutase (SOD), peroxidase (POD), and catalase (CAT) were determined following the method described by Wang et al. [20], with slight modifications.

Glutathione reductase (GR) facilitates the conversion of oxidized glutathione (GSSG) to its reduced form (GSH) by utilizing NADPH as an electron donor, resulting in the oxidation of NADPH to NADP^+^. NADPH exhibits a characteristic absorbance peak at 340 nm, whereas NADP^+^ does not absorb at this wavelength. Therefore, the rate of decrease in absorbance at 340 nm reflects the rate of NADPH oxidation, which is used to calculate GR activity.

### 2.5. Determination of Soluble Sugar, Soluble Protein, Proline (Pro), Hydrogen Peroxide (H_2_O_2_), and Malondialdehyde (MDA) Contents

The concentrations of soluble sugars, soluble proteins, and proline in the leaves of *A. nanchuanensis* seedlings were quantified using commercially available assay kits following the protocols provided by the manufacturer (Jiancheng Bioengineering Institute, Nanjing, China). H_2_O_2_ levels were assessed using a dedicated kit obtained from Solarbio (Beijing, China).

To evaluate the degree of lipid peroxidation, the MDA content was measured through the thiobarbituric acid (TBA) assay as described by Wang et al. [20]. Briefly, 0.3 mL of Reagent I was added to 0.1 mL of leaf extract in a 1.5 mL microcentrifuge tube, followed by thorough vortexing. The mixture was incubated at 95 °C for 30 min in a water bath with caps tightly sealed to avoid sample loss. After incubation, samples were rapidly cooled on ice and centrifuged at 10,000× *g* for 10 min at 25 °C. Then, 200 μL of the resulting supernatant was transferred into a 96-well plate, and absorbance values were recorded at 532 nm and 600 nm. The MDA concentration was calculated from the difference in absorbance (ΔA = A_532_ − A_600_).

### 2.6. Determination of Photosynthetic Pigment Content

About 0.1 g of fresh leaf tissue was finely chopped and incubated in 10 mL of 80% (*v*/*v*) acetone at room temperature in the dark for 24 h, allowing full pigment extraction. The absorbance of the resulting solution was then recorded at wavelengths of 663 nm, 645 nm, and 470 nm, using 80% acetone as the reference blank.

### 2.7. Determination of Ethylene and Its Biosynthetic Enzyme Concentrations

Leaf samples (0.1–0.5 g) were rinsed in pre-chilled phosphate-buffered saline (PBS, 0.05 mol·L^−1^, pH 7.4), blotted dry with filter paper, and accurately weighed. Samples were homogenized in nine volumes (*w*/*v* = 1:9) of ice-cold PBS using either a manual glass homogenizer (PYREX^®^ Dounce Tissue Grinder, Corning Inc., Corning, NY, USA) or an electric tissue grinder (Omni TH Tissue Homogenizer, Omni International, Kennesaw, GA, USA), under an ice bath to ensure protein stability. The homogenates were then centrifuged at 3000 rpm for 10–15 min at 4 °C, and the supernatants were collected for subsequent analysis.

The experimental method of Yuan et al. was appropriately modified, and the sample preparation and collection were completed [21]. The concentrations of ethylene (ETH), 1-aminocyclopropane-1-carboxylate synthase (ACS), 1-aminocyclopropane-1-carboxylate oxidase (ACO), and S-adenosylmethionine synthetase (SAMS) were determined using commercial enzyme-linked immunosorbent assay (ELISA) kits, following the manufacturer’s instructions (Nanjing Jiancheng Bioengineering Institute, Nanjing, China). Fresh samples (1 g) were ground with 10 mL of 0.1 M phosphate buffer solution (pH 7.4), and then centrifuged at 1000× *g* for 20 min at 4 °C. The supernatant was used for measurement. Concentrations were calculated according to the standard curve and converted to pmol·g^−1^ FW (ETH) or U·g^−1^ FW (ACS, ACO, SAMS) based on extract volume and sample fresh weight.

### 2.8. Transcriptome Sequencing and Data Analysis

Total RNA was isolated from the tissue using TRIzol^®^ Reagent, following the manufacturer’s protocol. RNA quality was assessed with a 5300 Bioanalyzer (Agilent Technologies, Santa Clara, CA, USA) and quantified using an ND-2000 spectrophotometer (NanoDrop Technologies, Wilmington, DE, USA). Only RNA samples meeting the following criteria were used for library construction: concentration ≥ 20 ng/μL, total RNA > 1 μg, and RQN > 4.5.

The RNA-seq transcriptome library for *A. nanchuanensis* was prepared using the Illumina^®^ Stranded mRNA Prep, Ligation kit (San Diego, CA, USA), starting with 1 μg of total RNA. In brief, messenger RNA was isolated through polyA selection using oligo (dT) beads and then fragmented using fragmentation buffer. Double-stranded cDNA was synthesized using random hexamer primers. Following synthesis, the cDNA underwent end-repair, phosphorylation, and adapter ligation according to the library construction protocol. The libraries were size-selected for cDNA fragments between 300 and 400 bp using magnetic beads, followed by PCR amplification for 10–15 cycles. After quantification with Qubit 4.0, sequencing was performed on a NovaSeq X Plus platform (PE150) using the NovaSeq Reagent Kit.

### 2.9. RNA Extraction and Quantitative Real-Time PCR (qRT-PCR)

Total RNA was isolated from *A. nanchuanensis* leaves using the Plant RNA Extraction Kit (TIANGEN, Beijing, China), and its concentration and purity were determined with a NanoDrop 2000 spectrophotometer (Thermo Fisher Scientific, Waltham, MA, USA). First-strand cDNA was synthesized using the PrimeScript RT reagent kit with gDNA Eraser (Takara, Dalian, China). Quantitative real-time PCR (qRT-PCR) was performed with TB Green Premix Ex Taq II (Takara), and fluorescence signals were measured on the CFX96 Real-Time PCR System (Bio-Rad, Laboratories, Hercules, CA, USA). Based on Zhang’s findings, we selected Tip2 as the internal reference gene [22], and relative expression levels were calculated using the 2^−ΔΔCt^ method [23]. Primers were designed using Primer Premier 6.0 (Supplementary Table S1).

### 2.10. Statistical Analysis

All experimental data were first tested for statistical assumptions, including normality, homogeneity of variance, and independence, followed by repeated-measures analysis to ensure analytical robustness. Analysis of variance (ANOVA), t-tests, and Duncan’s multiple range tests were performed using IBM SPSS Statistics 24.0 (IBM Corporation, Armonk, NY, USA) to evaluate differences among treatments (*p* < 0.05). To control for type I error in multiple comparisons, Bonferroni correction was applied, with the adjusted significance threshold defined as *p* < 0.05/m (m = number of comparisons). All results are presented as mean ± standard deviation (SD), and statistically significant differences are marked with asterisks in the figures.

For the transcriptomic data, genes with FPKM > 1 were subjected to weighted gene co-expression network analysis (WGCNA) in R (v1.68) using default settings [24]. The preliminary gene co-expression correlation matrix was transformed into an adjacency matrix by applying a soft-threshold power (β = 8). This adjacency matrix was then converted to a topological overlap matrix (TOM) using the dissimilarity measure method [25]. A hierarchical clustering tree was constructed based on the TOM dissimilarity coefficients, and gene modules were identified using the dynamic tree cut algorithm. Each branch of the clustering tree represents a distinct gene module. Physiological traits were incorporated as external phenotypic data for module–trait association analysis. The final WGCNA parameters were set as β power = 8, minModuleSize = 30, mergeCutHeight = 0.25, and minKMEtoStay = 0.3. All graphical outputs were generated using OriginPro 2018 (OriginLab, Northampton, MA, USA).

## 3. Results

### 3.1. Morphological Characteristics 

**Figure 1:**
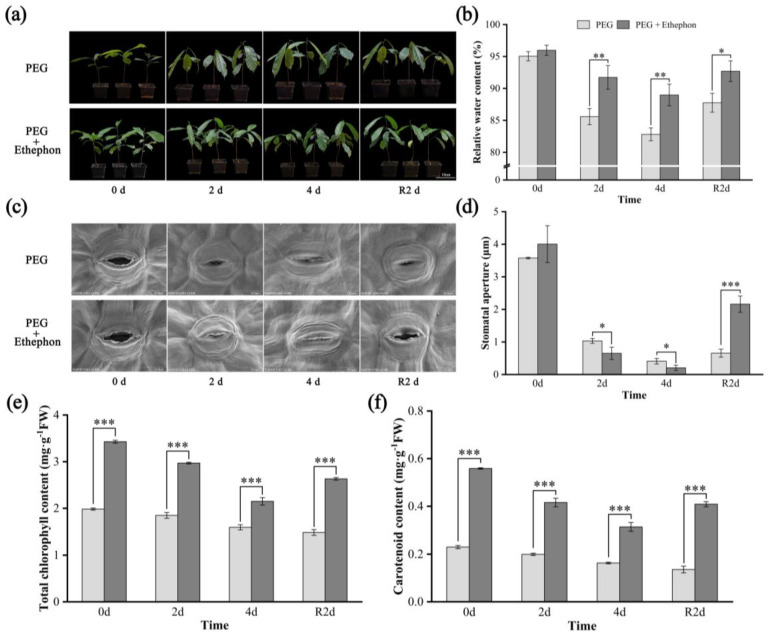
Morphological traits and physiological parameters of *A. nanchuanensis* seedlings after drought and rehydration. (**a**) Morphological traits of the PEG and PEG + Ethephon groups; (**b**) relative leaf water content; (**c**) stomatal imaging under scanning electron microscopy; (**d**) stomatal aperture; (**e**) total chlorophyll content; (**f**) carotenoid content. *, **, and *** indicate significant differences (*p* < 0.05), highly significant differences (*p* < 0.01), and extremely significant differences (*p* < 0.001), respectively, between the drought and ethylene groups.

Continuous monitoring of *A. nanchuanensis* seedlings under different drought treatments revealed distinct morphological responses (Figure 1a). With increasing duration of stress, seedlings in the PEG group exhibited more pronounced phenotypic changes compared to those in the PEG + Ethephon group. On day 4 of drought stress, severe morphological alterations were observed in the PEG-treated seedlings, including varying degrees of wilting in young and partially mature leaves, along with increased angles between petioles and branches, indicating turgor loss and leaf drooping. After 2 days of rehydration, water uptake resumed, and the condition of some young and mature leaves partially recovered; however, leaves that had undergone irreversible physiological damage failed to restore their original state. Overall, seedlings treated with PEG + Ethephon exhibited enhanced drought tolerance, as evidenced by milder phenotypic changes and consistently higher RWC in leaves across all time points compared to the PEG group (Figure 1b).

Stomata serve as key portals for gas exchange and play a fundamental role in plant photosynthesis. Microscopic observations of stomatal morphology in the leaves of *A. nanchuanensis* seedlings under drought stress (Figure 1c) revealed that pre-treatment with exogenous ethephon enhanced stomatal aperture under non-stress conditions (Figure 1d, 0 d), suggesting that ethephon application improves baseline gas exchange efficiency. As drought stress progressed, stomatal aperture in the PEG group gradually decreased, reaching 1.03 μm on day 2 and 0.41 μm on day 4. Following rehydration, aperture partially recovered to 0.66 μm. In contrast, the PEG + Ethephon group exhibited a more dynamic stomatal response. Apertures decreased to 0.54 μm and 0.24 μm on days 2 and 4, respectively, but significantly increased to 2.16 μm after 2 days of rewatering (R2 d), which was markedly higher than that of the PEG group. This indicates that ethephon not only modulates stomatal behavior during drought but also enhances the recovery capacity of *A. nanchuanensis* seedlings following rehydration (Figure 1d, R2 d).

Chlorophylls and carotenoids play critical roles in the photosynthetic apparatus of plants. To evaluate the impact of drought and subsequent rehydration on photosynthetic pigment accumulation in *A. nanchuanensis,* we quantified the contents of chlorophyll and carotenoids under different treatments. The results demonstrated that pre-application of exogenous ethephon effectively enhanced pigment accumulation during drought stress (Figure 1e,f). Both treatment groups showed a gradual decline in chlorophyll and carotenoid content with increasing drought duration. However, after rehydration, pigment levels increased in the PEG + Ethephon group (chlorophyll from 2.15 mg·g^−1^FW to 2.63 mg·g^−1^FW, carotenoid from 0.31 mg·g^−1^FW to 0.41 mg·g^−1^FW), while no recovery trend was observed in the PEG group (chlorophyll from 1.59 mg·g^−1^FW to 1.48 mg·g^−1^FW, carotenoid from 0.16 mg·g^−1^FW to 0.14 mg·g^−1^FW). Moreover, throughout the entire experimental period, photosynthetic pigment contents in the PEG + Ethephon group were significantly higher than those in the PEG group (*p* < 0.001). These findings indicate that ethephon not only improves chlorophyll and carotenoid levels under both normal and drought conditions but also facilitates the recovery of photosynthetic activity following rehydration.

### 3.2. Osmolyte, H_2_O_2_, and MDA Contents

**Figure 2:**
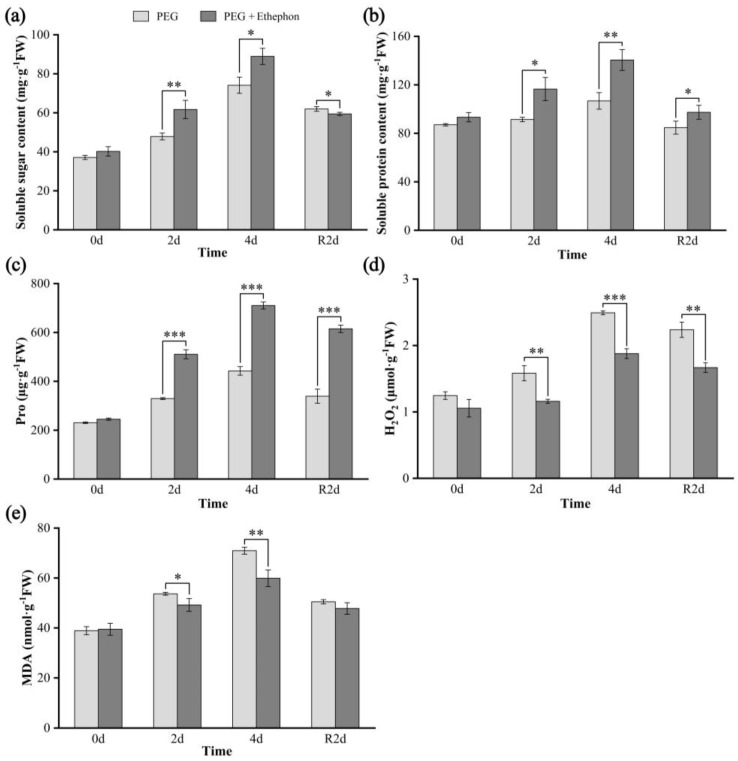
Changes in osmolytes and ROS markers in *A. nanchuanensis* under drought and rehydration treatments. (**a**) Soluble sugar; (**b**) soluble protein; (**c**) Pro; (**d**) H_2_O_2_; (**e**) MDA. *, **, and *** indicate significant differences (*p* < 0.05), highly significant differences (*p* < 0.01), and extremely significant differences (*p* < 0.001), respectively, between the drought and ethylene groups.

To further assess osmotic adjustment in *A. nanchuanensis* seedlings under drought stress, we quantified the levels of soluble sugars (Figure 2a), soluble proteins (Figure 2b), and proline (Figure 2c) in leaves. The results showed that the concentrations of all three osmolytes increased progressively with the duration of drought and declined after rehydration. Overall, the PEG + Ethephon group exhibited significantly or highly significantly higher levels of osmotic adjustment compounds across all time points compared to the PEG group, with the exception of soluble sugar content in the rehydration stage, where no significant difference was observed.

Under normal growth conditions, there were no noticeable differences in the levels of H_2_O_2_ and MDA between the two treatment groups. However, upon the onset of drought stress, the PEG group exhibited a sharp increase in both H_2_O_2_ and MDA contents with prolonged stress duration. On both day 2 and day 4 of drought treatment, H_2_O_2_ (*p* < 0.01 and *p* < 0.001) and MDA (*p* < 0.05 and *p* < 0.01) levels in the PEG group (H_2_O_2_: 1.58 μmol·g^−1^FW and 2.49 μmol·g^−1^FW, MDA: 53.68 μmol·g^−1^FW and 70.94 μmol·g^−1^FW) were significantly higher than those in the PEG + Ethephon group (H_2_O_2_: 1.16 μmol·g^−1^FW and 1.88 μmol·g^−1^FW, MDA: 49.19 μmol·g^−1^FW and 59.89 μmol·g^−1^FW) (Figure 2d,e). Although the PEG + Ethephon group showed a similar trend of increasing and then decreasing H_2_O_2_ and MDA levels during drought and rehydration, their overall concentrations remained lower and relatively more stable throughout the entire experimental period.

### 3.3. Antioxidant Enzyme Activities

**Figure 3:**
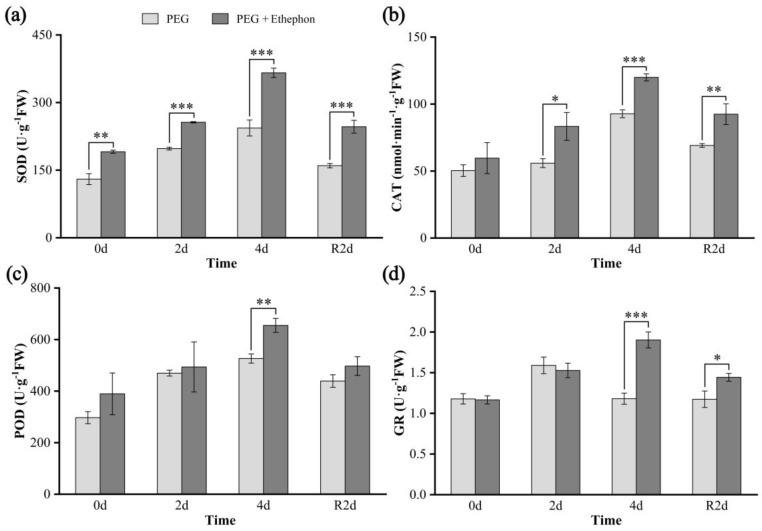
Changes in antioxidant enzyme activities in *A. nanchuanensis* seedlings under drought and rehydration treatments. (**a**) SOD; (**b**) CAT; (**c**) POD; (**d**) GR. *, **, and *** indicate significant differences (*p* < 0.05), highly significant differences (*p* < 0.01), and extremely significant differences (*p* < 0.001), respectively, between the drought and ethylene groups.

Exogenous application of ethephon enhanced the overall antioxidant enzyme activity in *A. nanchuanensis* seedlings. The activities of superoxide dismutase (SOD), catalase (CAT), and peroxidase (POD) were higher in the PEG + Ethephon group than in the PEG group across all time points, with highly significant differences (*p* < 0.01 and *p* < 0.001) observed on day 4 of drought stress (Figure 3a–c). Notably, glutathione reductase (GR) activity in the PEG group began to decline after two days of drought and gradually stabilized after rehydration. In contrast, GR activity in the PEG + Ethephon group continued to increase with prolonged drought stress and declined significantly after rehydration (Figure 3d). These results suggest that ethephon effectively promotes GR accumulation in *A. nanchuanensis*, thereby mitigating oxidative damage caused by drought stress.

### 3.4. Concentrations of Ethylene and Its Key Biosynthetic Enzymes

**Figure 4:**
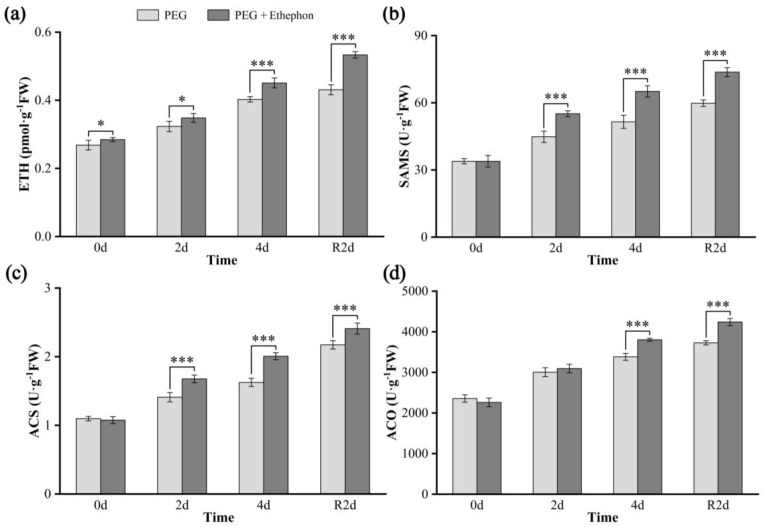
Changes in the concentrations of ethylene and key biosynthetic enzymes in *A. nanchuanensis* seedlings under stress and rehydration treatments. (**a**) ETH; (**b**) SAMS; (**c**) ACS; (**d**) ACO. * and *** indicate significant differences (*p* < 0.05) and extremely significant differences (*p* < 0.001), respectively, between the drought and ethylene groups.

With increasing drought duration, ethylene (ETH) content in the leaves of *A. nanchuanensis* seedlings gradually increased in both treatment groups. At all time points, ETH levels in the PEG + Ethephon group were significantly or highly significantly higher than those in the PEG group (Figure 4a). SAMS, ACS, and ACO are key enzymes involved in ethylene biosynthesis. Before the onset of drought stress, there were no significant differences in the levels of these enzymes between the two groups. However, by day 2 of drought stress, SAMS and ACS levels in the PEG + Ethephon group (55.11 U·g^−1^FW) were significantly higher (*p* < 0.001) than those in the PEG group (44.82 U·g^−1^FW), while ACO levels became significantly elevated (*p* < 0.001) on day 4 (Figure 4b–d). Notably, all three enzymes continued to increase even after two days of rehydration, maintaining relatively high levels.

### 3.5. Expression Patterns and Functional Annotation of DEGs

**Figure 5:**
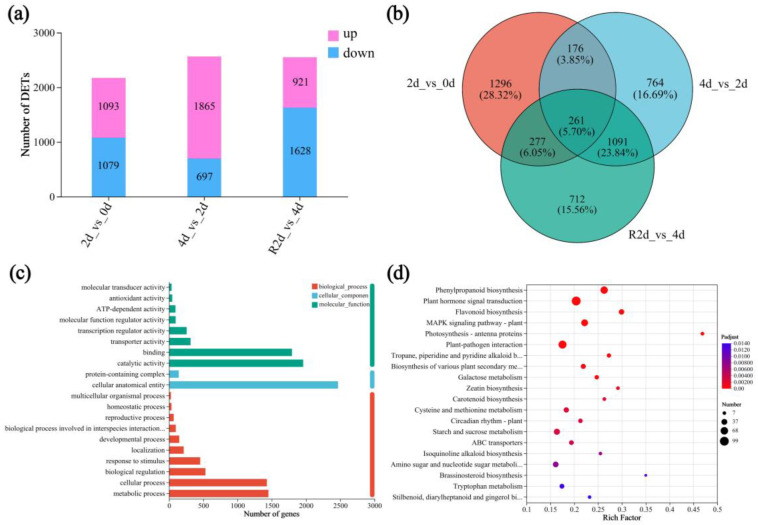
Comparison of DEGs among the three groups after drought and subsequent rewatering. (**a**) Stacked bar chart of DEGs. (**b**) Venn diagram; (**c**) GO annotation; (**d**) KEGG pathway enrichment. Here, 0 d to 4 d represent the duration of drought stress, and R2 d represents rehydration for 2 days. The 2 d vs. 0 d, 4 d vs. 2 d, and R2 d vs. 4 d comparisons refer to the three comparison groups. The size of each dot corresponds to the number of unique genes, while the color of the dot indicates the q-value.

The preceding results indicate that drought stress promotes ethylene biosynthesis in *A. nanchuanensis* leaves, and exogenous application of ethephon significantly enhances endogenous ethylene levels and the accumulation of ethylene’s key biosynthetic enzymes during stress. Seedlings treated with ethephon exhibited stronger drought tolerance, suggesting a potential close link between ethylene production and drought resistance in *A. nanchuanensis*. To further explore this relationship, we performed transcriptome sequencing on leaf samples from 24 drought-treated plants (six biological replicates per time point; see Supplementary Table S2 for sequencing statistics). Differentially expressed genes (DEGs) were identified using three pairwise comparisons: 2 d vs. 0 d, 4 d vs. 2 d, and R2 d vs. 4 d. The results showed a progressive increase in the number of upregulated genes with drought duration, reaching 1865 on day 4, while the number of downregulated genes decreased to 697. Following rehydration, the number of downregulated genes sharply increased to 1628, whereas upregulated genes decreased to 921 (Figure 5a). A Venn diagram analysis revealed that 261 DEGs were shared across all three comparisons, representing 5.70% of the total DEGs (Figure 5b). The comparison with the highest number of DEGs was R2 d vs. 4 d (2341), followed by 4 d vs. 2 d (2292) and 2 d vs. 0 d (2010).

Gene Ontology (GO) annotation classified the DEGs into three main categories: molecular function (MF), cellular component (CC), and biological process (BP), comprising 4599, 2610, and 4458 DEGs, respectively (Figure 5c). Within the MF category, catalytic activity and binding were the dominant terms, accounting for 81.63% of the total annotated unigenes. In the CC category, cellular anatomical entity was the largest group, representing 94.56% of the total unigenes. In the BP category, cellular process and metabolic process together accounted for 64.60% of the annotated unigenes.

Based on the KEGG enrichment analysis, the top 20 significantly enriched metabolic pathways among the total DEGs were visualized using a bubble plot (Figure 5d). Notably enriched pathways included those involved in soluble sugar accumulation (starch and sucrose metabolism), ROS detoxification systems (phenylpropanoid biosynthesis and plant hormone signal transduction), abscisic acid (ABA) metabolism (carotenoid biosynthesis and plant hormone signal transduction), and ethylene-related signaling (MAPK signaling pathway—plant).

### 3.6. Expression Profiles of the Ethylene Biosynthesis Pathway and Its Key Genes

**Figure 6:**
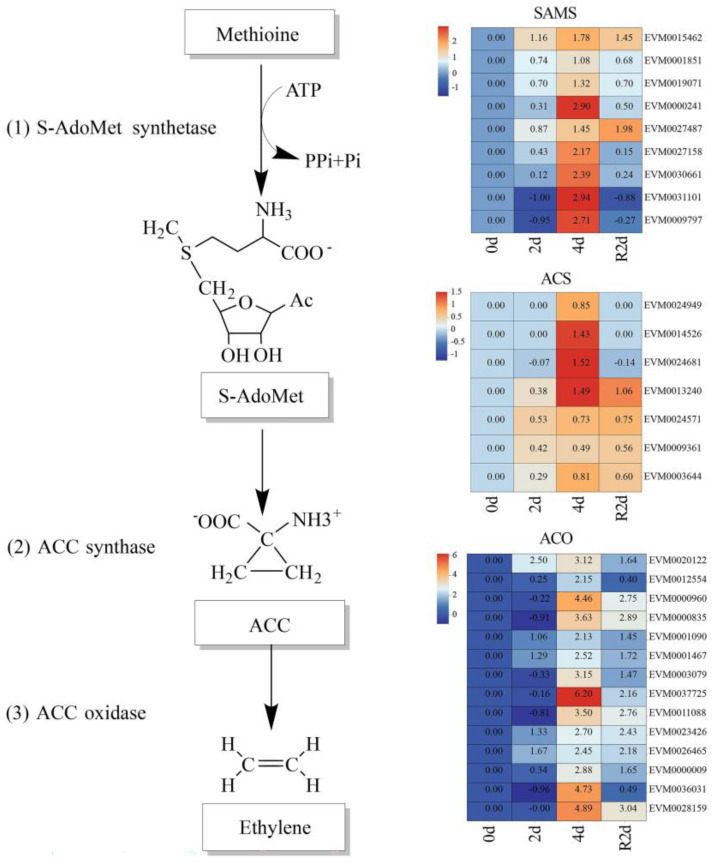
Ethylene biosynthesis pathway and its key genes. SAMS, ACS, and ACO are key enzymes involved in ethylene biosynthesis.

To elucidate the role of ethylene in the drought response of *A. nanchuanensis*, we analyzed the expression profiles of key ethylene biosynthesis genes among the DEGs (Figure 6). The results showed that the expression levels of genes involved in the biosynthetic pathways of SAMS, ACS, and ACO were markedly upregulated under drought stress and continued to increase with prolonged drought duration. After rehydration, their expression levels declined significantly. These findings indicate that the expression of ethylene biosynthesis genes is strongly induced by drought stress, thereby enhancing ethylene production and contributing to improved drought tolerance in *A. nanchuanensis* seedlings.

To validate the gene expression patterns obtained from RNA-Seq data, five key enzyme genes involved in the ethylene biosynthesis pathway were chosen for qRT-PCR analysis. The qRT-PCR results exhibited a strong positive correlation with the FPKM values from the RNA-Seq data, verifying the accuracy of the transcriptome findings (see Supplementary Figure S1).

### 3.7. Expression and Screening of Drought-Resistance Genes

**Figure 7:**
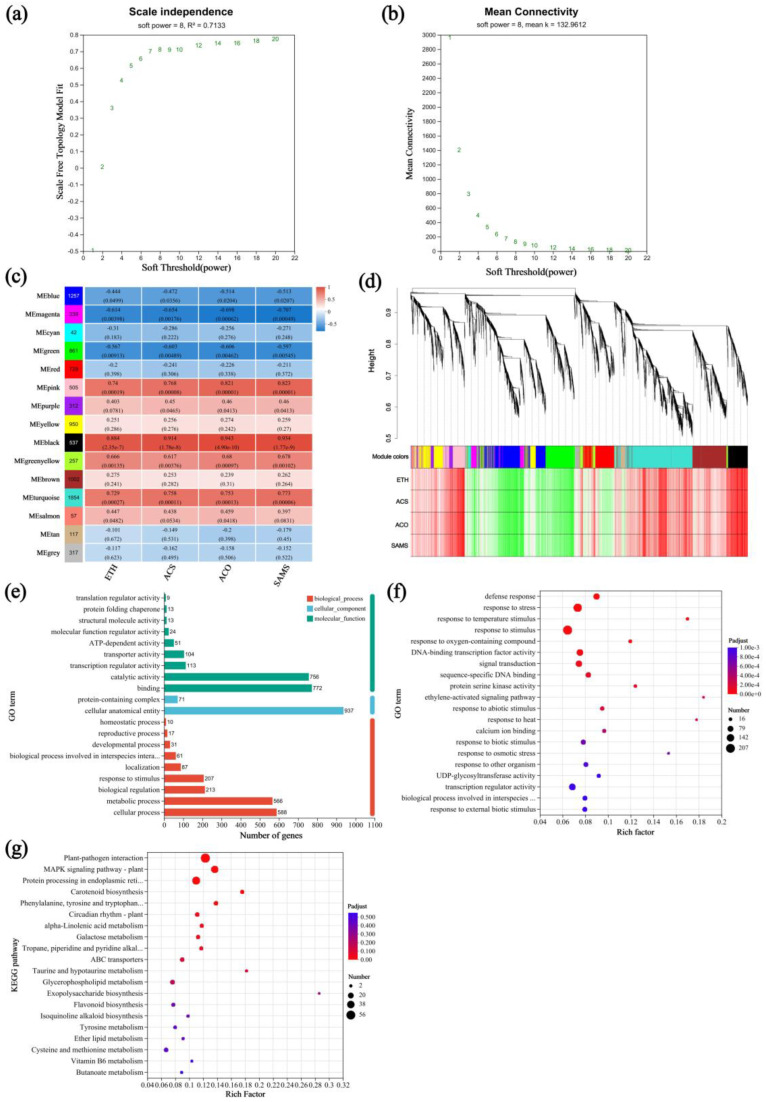
WGCNA of ethylene-biosynthesis-related genes. (**a**) Scale-free topology fit curve; (**b**) scale-free mean connectivity curve; (**c**) correlations between co-expression modules and physiological traits; (**d**) hierarchical clustering dendrogram showing 15 gene co-expression modules; (**e**) GO annotation of the MEturquoise module; (**f**) GO enrichment analysis of the MEturquoise module; (**g**) KEGG pathway enrichment analysis of the MEturquoise module.

To identify key genes involved in the drought response of *A. nanchuanensis*, ethylene and its key biosynthetic enzymes under drought and rehydration conditions were used as phenotypic traits for weighted gene co-expression network analysis (WGCNA). After data preprocessing, the correlation strength among genes was adjusted to fit a scale-free topology (Figure 7a,b). A total of 9136 unigenes were subjected to WGCNA, resulting in their classification into 15 distinct modules (Figure 7c,d). Among these, the MEturquoise module contained the highest number of unigenes (1854), while the MEcyan module had the fewest (42). Using a correlation coefficient threshold of r > 0.7 between modules and phenotypic traits, the MEturquoise module was found to be not only the highest but also significantly positively correlated with ETH (r = 0.729, *p* = 0.00027), ACS (r = 0.758, *p* = 0.00011), ACO (r = 0.753, *p* = 0.00013), and SAMS (r = 0.773, *p* = 0.00006). These results suggest that genes within the MEturquoise module are closely associated with ethylene-mediated drought tolerance in *A. nanchuanensis* seedlings. Therefore, this module was selected for further exploration of candidate genes.

GO annotation of the MEturquoise module revealed significant enrichment in key functional categories (Figure 7e), including cellular process (GO:0009987) and metabolic process (GO:0008152) in the BP category, binding (GO:0005488) and catalytic activity (GO:0003824) in the MF category, and cellular anatomical entity (GO:0110165) in the CC category. Further functional enrichment analysis based on both GO and KEGG (Figure 7f,g) identified the top 20 significantly enriched pathways (*p* < 0.05), visualized in bubble plots. The genes within the MEturquoise module were highly enriched in stress-related pathways, including response to stress, response to stimulus, MAPK signaling pathway—plant, and protein processing in endoplasmic reticulum. Notably, two genes encoding *MPK3* (EVM0025051 and EVM0041510) were identified in the MAPK signaling pathway and were predicted to be involved in ethylene signal transduction. In addition, 42 genes from this module were associated with the protein processing in endoplasmic reticulum pathway, among which six were identified as heat shock protein (HSP)-encoding genes: three *HSP70s* (EVM0035899, EVM0011013, and EVM0019315), two *HSP90s* (EVM0005362 and EVM0038963), and one *HSP40* (EVM0022390). GO enrichment analysis further revealed that the three *HSP70* genes were also annotated under the response to stress category. These results suggest that the above-mentioned genes may play important roles in drought tolerance in *A. nanchuanensis* seedlings. To validate their expression patterns, five representative genes were randomly selected for qRT-PCR analysis (see Supplementary Figure S1). The relative expression levels obtained by qRT-PCR were significantly positively correlated with FPKM values from RNA-Seq, confirming the reliability of the transcriptomic data.

The size of each dot corresponds to the number of unique genes, while the color of the dot indicates the q-value.

## 4. Discussion

### 4.1. Phenotypic Responses of A. nanchuanensis to Drought Stress

Drought stress profoundly influences the morphology, anatomy, stomatal dynamics, and photosynthetic performance of woody plants and is widely recognized as a core driver of drought adaptation. RWC is a reliable indicator of plant water status, and numerous studies have reported significant RWC declines in woody plants [26]. Leaf wilting is among the earliest visible dehydration symptoms, preceding irreversible cellular damage [27]. Stomata, as the primary regulators of transpiration and gas exchange, respond rapidly in the early drought phase by closing to reduce water loss, leading to marked decreases in stomatal aperture [28,29].

The reduction in photosynthesis under drought results from both stomatal and non-stomatal limitations. Stomatal closure restricts CO_2_ uptake, thereby lowering photosynthetic efficiency, while non-stomatal factors—such as pigment degradation and chloroplast damage—further constrain photosynthetic capacity [30]. Chlorophyll and carotenoids serve as critical indicators of both photosynthetic stability and antioxidant potential. While drought typically accelerates chlorophyll degradation, genotypes maintaining higher chlorophyll levels tend to preserve photosynthetic rates [31]. Carotenoids, key antioxidants and membrane stabilizers, not only scavenge ROS but also reinforce thylakoid and cellular membrane integrity [32,33,34,35,36]. Their synthesis and regulation vary among species and depend on drought severity and duration.

Ethylene plays a distinct role in modulating stomatal behavior during drought, particularly when applied exogenously as ethephon. Ethylene-induced stomatal closure involves ROS and downstream signaling components such as EIN2 and EIN3 [37,38]. In some woody species (e.g., *Fraxinus chinensis*), stomata fail to reopen efficiently after rehydration, and antagonists such as 1-MCP can accelerate reopening, suggesting that ethylene inhibits stomatal recovery during rehydration [39]. Other studies have shown that exogenous ethylene accelerates stomatal closure via ROS, NO, and/or H_2_S signaling and can sustain low stomatal conductance during rehydration even after ABA declines [40].

In this study, ethephon application under non-stress conditions enhanced stomatal conductance and increased chlorophyll and carotenoid contents in *A. nanchuanensis*, resulting in improved photosynthetic efficiency. Under PEG-induced drought, pigment contents in the PEG + Ethephon group were 1.5 and 2 times higher, respectively, than those in the PEG group, and remained significantly elevated across all drought stages (Figure 1e,f). This indicates that exogenous ethephon not only promotes photosynthesis in well-watered seedlings but also helps maintain pigment stability under drought. Upon rehydration, pigment levels and photosynthetic activity recovered markedly in the PEG + Ethephon group, whereas recovery was negligible in the PEG group. Furthermore, ethephon-treated seedlings exhibited faster stomatal closure under drought while maintaining higher RWC, followed by rapid stomatal reopening and photosynthetic recovery after rehydration (Figure 1b–d). These results align with previous findings [41,42] and underscore the novel role of ethylene in sustaining photosynthetic stability and facilitating post-drought recovery in a critically endangered woody species.

### 4.2. Effects of Drought on Osmolytes and ROS Markers in A. nanchuanensis

Carbohydrates serve not only as the primary energy source for plants but also as key osmotic regulators that help maintain cellular water balance under water-deficit conditions [43]. Under drought stress, plants accumulate soluble sugars to lower cellular osmotic potential, thereby preventing dehydration and cell shrinkage. In addition to osmotic regulation, soluble sugars contribute to oxidative stress mitigation by scavenging excess ROS [44]. Soluble proteins also increase during drought, primarily due to the synthesis of stress-related enzymes, including antioxidant and osmolyte-regulating enzymes [45]. Proline is among the most effective osmotic regulators in plants; its accumulation improves cellular water retention, serves as an energy and respiratory substrate under nutrient limitation, and participates in chlorophyll biosynthesis [46]. H_2_O_2_ and MDA are widely used as oxidative stress markers in drought studies. H_2_O_2_ functions as a signaling molecule, activating the antioxidant defense system, while MDA, a lipid peroxidation byproduct, indicates the extent of membrane damage [47]. Drought stress commonly increases H_2_O_2_ and MDA contents, exacerbating oxidative damage and impairing plant growth and physiology [48]. Thus, quantifying these markers is crucial for assessing drought-induced oxidative stress and underlying tolerance mechanisms.

In this study, *A. nanchuanensis* seedlings in the PEG + Ethephon group exhibited consistently higher levels of osmotic adjustment substances (soluble sugars, soluble proteins, and proline) than the PEG group during both drought and rehydration phases, with significant or highly significant differences at all stages (Figure 2a–c). This suggests a greater ROS-scavenging capacity in the PEG + Ethephon group. Although levels of osmolytes declined after two days of rehydration in both treatments, the PEG + Ethephon group maintained a significant advantage.

Drought stress also led to marked increases in H_2_O_2_ and MDA levels, with both parameters being positively correlated with drought duration and decreasing upon rehydration (Figure 2d,e). At all stages except 0 days, the PEG + Ethephon group maintained lower H_2_O_2_ and MDA levels than the PEG group, indicating reduced oxidative membrane damage.

Overall, these results suggest that exogenous ethephon promotes the synthesis of osmotic regulators in *A. nanchuanensis*, thereby sustaining root water uptake and enhancing cellular water retention under drought stress, ultimately improving drought tolerance in seedlings of this critically endangered species.

### 4.3. Effects of Drought on the Antioxidant System of A. nanchuanensis

Drought imposes severe osmotic stress on plants, disrupting intracellular redox homeostasis and inhibiting growth [49]. ROS, including singlet oxygen, superoxide anions, hydroxyl radicals, and H_2_O_2_, are highly reactive oxygen-containing molecules and their derivatives [50]. As key intracellular signaling molecules, ROS levels fluctuate in response to environmental changes but are normally maintained within a physiological range. Excessive ROS accumulation can damage cellular structures and impair metabolic functions, whereas insufficient ROS can also hinder growth and development [51,52]. Under drought stress, plants enhance the activities of antioxidant enzymes (such as SOD, POD, and CAT) to scavenge excess ROS and mitigate oxidative injury [53,54]. These enzymes act in different subcellular compartments and coordinate synergistically to maintain redox balance [55].

In this study, SOD, CAT, and POD activities were consistently higher in the PEG + Ethephon group than in the PEG group during both drought and rehydration phases, with differences becoming more pronounced as drought progressed (Figure 3a–c). GR catalyzes the reduction of oxidized glutathione (GSSG) to its reduced form (GSH), thereby sustaining a high GSH/GSSG ratio essential for antioxidant capacity [56]. Given the pivotal role of GSH in ROS detoxification, GR activity directly influences the efficiency of the antioxidant defense system [57]. In the PEG group, GR activity in *A. nanchuanensis* seedlings initially increased under drought, followed by a decline with prolonged stress (Figure 3d), and then remained relatively stable after rehydration. This pattern aligns with previous findings that early ROS accumulation triggers GR gene upregulation and enzyme activation to preserve the GSH/GSSG ratio [58]. However, sustained drought—accompanied by reduced leaf relative water content—may limit NADPH (Nicotinamide Adenine Dinucleotide Phosphate) availability and protein repair capacity, leading to oxidative inactivation of GR via carbonylation or S-nitrosylation [59,60,61].

In contrast, GR activity in the PEG + Ethephon group continued to increase throughout the drought period, suggesting that photosynthesis or oxidative phosphorylation may still supply sufficient NADPH under ethephon treatment. This sustained GR activity implies that exogenous ethephon can enhance antioxidant enzyme function and ROS-scavenging efficiency, thereby improving oxidative stress tolerance in *A. nanchuanensis* seedlings [62,63].

### 4.4. Effects of Drought on Ethylene Production and Key Biosynthetic Enzymes in A. nanchuanensis

Extensive research has shown that moderate drought stress elevates ethylene levels in plants, primarily through increased concentrations of key biosynthetic enzymes such as SAMS, ACS, and ACO. In contrast, under severe or prolonged drought, ACS and ACO concentrations often decline, thereby restricting ethylene production [64,65]. Drought typically triggers a rapid rise in SAMS concentration at the early stage, providing SAM as a substrate for subsequent ACC synthesis [66]. The ethylene peak produced through the ACS–ACO cascade generally occurs 6–24 h before the abscisic acid (ABA) peak, activating the EIN3/EIL–ERF transcriptional module. This activation induces a range of stress-responsive genes, such as P5CS (involved in proline biosynthesis) and CAT2 (involved in ROS detoxification) [67,68], underscoring the central role of ethylene and its biosynthetic enzymes in conferring abiotic stress tolerance.

In this study, under non-stress conditions, exogenous ethephon application markedly increased endogenous ethylene content in *A. nanchuanensis* seedlings (Figure 4a, 0 d), whereas the concentrations of SAMS, ACS, and ACO showed no significant differences between treatments at this stage (Figure 4b–d, 0 d). This suggests that the elevated ethylene levels under well-watered conditions primarily resulted from the direct release of ethylene through ethephon decomposition, rather than from enhanced endogenous biosynthesis.

Once drought stress commenced, the levels of ethylene biosynthetic enzymes increased rapidly in both treatment groups, accompanied by a corresponding rise in ethylene content. As drought progressed, ETH, SAMS, ACS, and ACO levels continued to increase, with the PEG + Ethephon group consistently exhibiting significantly or highly significantly higher values compared with the PEG group. These results indicate that under drought stress, ethephon application not only elevates ethylene levels via decomposition but also stimulates substantial upregulation of SAMS, ACS, and ACO, thereby enhancing endogenous ethylene biosynthesis and strengthening drought response in *A. nanchuanensis*.

### 4.5. Effects of Drought on the Transcriptome of A. nanchuanensis

Extensive genetic and physiological evidence has established ethylene and its key biosynthetic enzymes, ACS and ACO, as pivotal signaling hubs within the plant drought response network. During the early stages of drought, ethylene biosynthetic genes are rapidly upregulated, with upstream *SAMS* and downstream *ACS* and *ACO* genes being synchronously induced. This leads to the initial accumulation of ACC and ethylene, facilitating rapid stomatal closure and ROS scavenging [68,69]. In *Petunia*, disruption of either *PhACO1* or *PhACO3* significantly reduced ethylene levels in mutant leaves, resulting in decreased tolerance to drought and salinity compared to the wild type [68]. Similarly, silencing *GhSAMS2* via virus-induced gene silencing (VIGS) in cotton led to heightened sensitivity to both drought and salt stress [66]. These findings underscore the essential role of ethylene-biosynthesis-related genes in modulating drought resistance and highlight their potential as key targets for enhancing plant stress resilience.

In this study, we demonstrated that exogenous ethephon application enhanced drought tolerance in *A. nanchuanensis* seedlings. To further investigate the role of endogenous ethylene in drought response, we conducted transcriptome sequencing on leaf samples from PEG-treated seedlings. The results revealed a strong association between the drought tolerance of *A. nanchuanensis* and its endogenous ethylene biosynthesis. Specifically, genes encoding ethylene biosynthetic enzymes, including *SAMS*, *ACO*, and *ACS*, were found to play critical roles in enhancing drought resistance (Figure 6). Expression levels of several key genes significantly increased with prolonged drought stress, indicating their potential contribution to improving the drought tolerance of *A. nanchuanensis* seedlings. Furthermore, WGCNA analysis identified eight key drought-resistance genes associated with the ethylene signaling pathway. Numerous studies have shown that the molecular chaperone network composed of *HSP90*, *HSP70*, *HSP40*, and the *MPK3* signaling module supports drought tolerance through two key mechanisms: “protein homeostasis or ROS scavenging” and “stomatal rapid regulation or signal amplification”. These mechanisms are interconnected through the ROS node and hormonal interactions (ABA, ethylene, or SA), forming a synergistic defense [70,71,72,73,74,75]. *HSP90*, *HSP70*, *HSP40*, and the *MPK3* candidate genes identified in this study are likely key nodes in the “chaperone homeostasis–ROS regulation–stomatal signaling” network. Functional validation of these genes (e.g., overexpression or CRISPR knockout, protein interactions, and phosphorylation site identification) may elucidate their specific roles in the “ethylene–ROS–stomatal or pigment–osmotic regulation” pathway, thereby providing valuable targets for molecular improvement in drought resistance of critically endangered tree species.

## 5. Conclusions

This study demonstrates that drought stress in *A. nanchuanensis* seedlings reduces relative water content and photosynthetic pigment levels, thereby impairing photosynthetic efficiency and inducing oxidative damage. These effects intensified with prolonged stress and were only partially alleviated after rehydration. Drought stress also stimulated endogenous ethylene biosynthesis, as evidenced by the increased concentrations of SAMS, ACS, and ACO in PEG-treated seedlings.

Exogenous application of ethephon significantly enhanced ethylene accumulation and further increased the activity of key biosynthetic enzymes under drought stress. Compared with the PEG group, ethephon-treated seedlings maintained higher relative water content, photosynthetic pigment levels, and osmotic adjustment substance contents, as well as stronger antioxidant capacity and photosynthetic efficiency, with a more pronounced recovery after rehydration. These results confirm that ethylene is a central hormone in enhancing drought tolerance and post-stress recovery in *A. nanchuanensis*, and that exogenous ethephon application is an effective strategy for improving drought resilience in this critically endangered species.

At the molecular level, transcriptome analysis under drought stress revealed the ethylene biosynthesis pathway in *A. nanchuanensis* and identified key drought-responsive genes, including *SAMS*, *ACS*, and *ACO*. Weighted gene co-expression network analysis (WGCNA) further identified eight candidate drought-resistance genes—two *MPK3* genes, three *HSP70s*, two *HSP90s*, and one *HSP40*—which represent promising targets for functional characterization and molecular breeding. These findings provide a valuable genetic resource for advancing the conservation and genetic improvement of *A. nanchuanensis*.

## 6. Limitations and Future Directions

This study is limited by its sample size and the use of PEG-induced drought rather than natural drought conditions. Future work should expand sample numbers and incorporate field-based experiments to better capture natural environmental variability. In addition, establishing a stable genetic transformation system for *A. nanchuanensis* will be critical for functional and structural characterization of the identified key drought-related genes, thereby accelerating the application of molecular breeding and improving conservation strategies for this endangered species.

## Data Availability

The original contributions presented in this study are included in the article/Supplementary Material. Further inquiries can be directed to the corresponding author.

## Supplementary Materials

The following supporting information can be downloaded at: https://www.mdpi.com/article/10.3390/plants14172636/s1, Table S1: Primers used for qRT-PCR of key drought-responsive genes; Table S2: Summary of transcriptome sequencing data for 24 *A. nanchuanensis* samples subjected to drought stress; Figure S1: qRT-PCR analysis of key drought-responsive genes.
