# Supplementary material for: Ethylene-Mediated Drought Tolerance in the Critically Endangered Artocarpus nanchuanensis: Insights from Physiological and Transcriptomic Analyses
Source: Plants (Basel). 2025 Aug 24;14(17):2636. doi: 10.3390/plants14172636 (PMC12430093; doi:10.3390/plants14172636)
Supplement: Supplementary file 1 [file plants-14-02636-s001.zip › plants-3805838-supplementary/Tables S1 and S2.pdf]

Supplementary Table S1 Primers used for qRT-PCR of key drought-responsive genes.

Gene	Primer sequence (5'-3')	Gene ID
SAMS1	F 5′-AAGAGTGTGGTGGCTTCTGG-3′<br>R 5′-CCCGTCTTGTAGGTGTCAACA-3′	EVM0000241
SAMS2	F 5′-ATTGATGAGGGACGGCTTGG-3′<br>R 5′-CCTGGCTCTACCTTCATGTTCA-3′	EVM0027158
SAMS3	F 5′-CGAACCCTGTGGACGTGATC-3′<br>R 5′-GAAGCCCTTGTAGAGCGACAT-3′	EVM0030661
ACS1	F 5′-GTGACAAAGGCAAAGCGGAAT-3′<br>R 5′-GAGGCATAGGTGAACGAGGAG-3′	EVM0014526
ACO3	F 5′-GCCAGAGGATATAATCGCACCA-3′<br>R 5′-CCATCGTGAGCCTTCCTGTAG-3′	EVM0037725
HSP90a	F 5′-ACAGGATGTTGAAGCTTGGTCT-3′<br>R 5′-CAACCTCCTCCATCTTGCTCTC-3′	EVM0005362
HSP90b	F 5′-GAAACTGCCCTGTTGACCTCT-3′<br>R 5′-TCCATGTTGCTCTCCTCATTCC-3′	EVM0038963
HSP40a	F 5′-GAGGAGACAACTCTGCACGAT-3′<br>R 5′-CACTCTCTGAGCACCACCAT-3′	EVM0022390
MPK3a	F 5′-GCATGATGTTGCTGATGAACCA-3′<br>R 5′-TTGAGTGCAATGGCCTCCTG-3′	EVM0025051
MPK3b	F 5′-GCATGATGTTGCTGATGAACCA-3′<br>R 5′-GAATGCAATGGCCTCCTGGA-3′	EVM0041510
Tip2	F 5′-TGGCTTAGAGTTGATGGAGTGC-3′<br>R 5′-TAGGAAGCGGAGTCGGTACG-3′	

Supplementary Table S2 Summary of transcriptome sequencing data for 24 *A. nanchuanensis* samples subjected to drought stress.

Sample	Clean reads	Clean bases	Q30	GC content	Mapped reads	Unique match
0d-1	50546766	7527590661	95.49%	45.1%	94.88%	91.62%
0d-2	47174678	7016527685	95.38%	45.01%	94.84%	91.03%
0d-3	46724710	6964546236	95.37%	45.01%	94.73%	91.26%
0d-4	45566550	6799341992	95.08%	45.11%	94.84%	91.50%

0d-5	55332656	8246270539	95.55%	45.04%	94.80%	91.31%
0d-6	47476864	7077916017	95.40%	44.96%	94.69%	91.20%
2d-1	43392240	6491385101	95.52%	45.13%	94.76%	90.87%
2d-2	50804152	7574336590	95.33%	45.13%	94.69%	91.01%
2d-3	45789250	6832231505	95.42%	45.17%	94.6%	91.59%
2d-4	47030884	7014219460	95.26%	45.09%	94.68%	91.34%
2d-5	50972964	7604190886	95.36%	45.28%	94.84%	91.82%
2d-6	52785870	7866046471	95.49%	45.11%	94.81%	91.12%
4d-1	50493114	7543460892	95.32%	44.79%	94.52%	91.26%
4d-2	50872288	7571275058	95.34%	44.99%	94.71%	91.63%
4d-3	56258012	8387921905	95.44%	44.86%	94.60%	91.71%
4d-4	48302916	7214195186	95.46%	44.84%	94.62%	91.64%
4d-5	52979734	7907773084	95.53%	45.10%	94.73%	91.62%
4d-6	51676970	7701563449	95.39%	45.01%	94.78%	91.55%
R2d-1	53579922	7985089300	95.45%	44.82%	94.83%	91.66%
R2d-2	46201420	6894288504	95.29%	44.66%	94.66%	91.46%
R2d-3	45232518	6754915271	95.37%	44.65%	94.75%	91.67%
R2d-4	56539562	8432738584	95.54%	44.60%	94.69%	91.52%
R2d-5	48850362	7287170183	95.62%	44.89%	94.84%	91.77%
R2d-6	47850180	7137732862	95.15%	44.84%	94.67%	91.30%
